# Supplementary material for: Direct Antiviral Treatments for Hepatitis C Virus Have Off-Target Effects of Oncologic Relevance in Hepatocellular Carcinoma
Source: Cancers (Basel). 2020 Sep 19;12(9):2674. doi: 10.3390/cancers12092674 (PMC7565876; doi:10.3390/cancers12092674)
Supplement: Supplementary file 1 [file cancers-12-02674-s001.pdf]

cancers

MDPI

Article

Direct Antiviral Treatments for Hepatitis C Virus Have Off-Target Effects of Oncologic Relevance in Hepatocellular Carcinoma

Catia Giovannini, Francesca Fornari, Valentina Indio, Davide Trerè, Matteo Renzulli, Francesco Vasuri, Matteo Cescon, Matteo Ravaioli, Alessia Perrucci, Annalisa Astolfi, Fabio Piscaglia and Laura Gramantieri

1. Supplementary Materials and Methods

1.1. Cell Proliferation Assay

Crystal violet assay was used for a quick and reliable determination of cell viability. Cells were seeded in 24-wells plate and treated with DAAs at a concentration of 4 µM for Sofosbuvir and 10 nM for Daclatasvir for 24 and 48 h [1]. Control cells were treated with DMSO concentrations reflecting those used in each drug experiment. Cell staining with crystal violet and spectrophotometric quantification was previously described [2]. Assays were performed in triplicate.

1.2. Cell Cycle Analysis

FACS analysis was performed to define changes in cell cycle distribution resulting from DAA treatments in HCC-derived cell lines. Cells were synchronized, seeded in 6-well plates and treated with DAAs at previously reported concentrations, 4 µM for Sofosbuvir and 10 nM for Daclatasvir [1]. Twenty-four hours post DAAs treatment, adherent cells were collected and fixed with 70% ethanol and cell cycle analysis was performed using Cytoflex S (Beckman Coulter, Brea, CA, USA).

1.3. Cell Plating and Scratching

Cell motility and migration are hallmarks of cancer cells. The scratch assay was used here to gain information on any variation of cell migration capability occurring after DAA treatments. Huh7, SNU475, SNU449, HepG2, Hep3B, TFK1, Huh28, LnCAP and MCF7 cells were seeded in 96-well plate. After reaching confluence, a uniform and reproducible scratch was made using a WoundMaker™ (Essen BioScience, Ann Harbor, MI, USA). After creating the scratch, the medium was aspirated, and the wells washed and replenished with fresh medium supplemented with 1% FBS and containing DAAs or DMSO at concentrations matching those used in the corresponding treated arm. Cell cultures were analysed by the IncuCyte live-cell imaging system (Essen BioScience) set up to take pictures of cells every 3 h. The imaging system measures the closed area of the scratch in real time and automatically calculates the cell area, scratch width, and cell density percentage at each time point.

1.4. Real-Time qPCR

For validation purposes of gene expression changes, cell lines treated by sofosbuvir and daclatasvir were examined for HIST1H3A, HIST1H4A expression by quantitative PCR (qPCR) as previously described [3]. The same analyses were performed in HCC and cirrhotic tissues. GAPDH housekeeping gene was considered for gene normalization. QPCR experiments were run in triplicate. Primers and conditions are detailed in Table S1.

1.5. Immunohistochemistry

The expression of FOXM1 (Novus Biologicals, Centennial, CO, USA), VIM (Cell Signaling, Danvers, MA, USA) and Ki67 (Dako, Glostrup, Denmark) Labelling Index (LI) in HCC tissues were immunohistochemically assessed on formalin-fixed, paraffin-embedded sections as previously described [4]. Negative controls were done by omitting the primary antibody. Immunoreactivity was revealed with the EnVision system (Dako), and diaminobenzidine (DAB) as chromogen (Sigma, St. Louis, MO, USA). Slides were counterstained in Meyer's hematoxylin, coverslipped and examined by light microscopy. VIM and FOXM1 immunostaining was assessed on 20 consecutive 40X magnification fields by two independent observers (C.G., L.G.) using a validated semi-quantitative scale and categorizing cases in three groups: staining of <5% cells; staining of 5–30% cells; staining of >30% cells. Ki-67 staining was quantified by image cytometry using Image J software (NIH, Bethesda, MD, USA) on at least 10 randomly selected fields at 40X magnification and expressed as the percentage of positive nuclei over the total nuclei (Labeling index:LI).

Table S1. Primer sequences and qPCR conditions.

Gene	Primer Sequences	Amplified Product (bp)	Annealing T (°C)	Cycles (N.)
HIST1H3A	Fw 5′-CTTGGTAGGGCTATTTGAGGAC-3′<br>Rv 5′-TTTGCTTGGACCGTCAGAG-3′	142	61.8	45
HIST1H4A	Fw 5′-CTGTCACCTATACGGAGCAC-3′<br>Rv 5′-GGGCCTTTGGTTCAGAAATG-3′	147	61.8	45
GAPDH	Fw 5′-ACATCGCTCAGACACCATG-3′<br>Rv 5′-TGTAGTTGAGGTCAATGAAGGG-3′	87	61.8	40

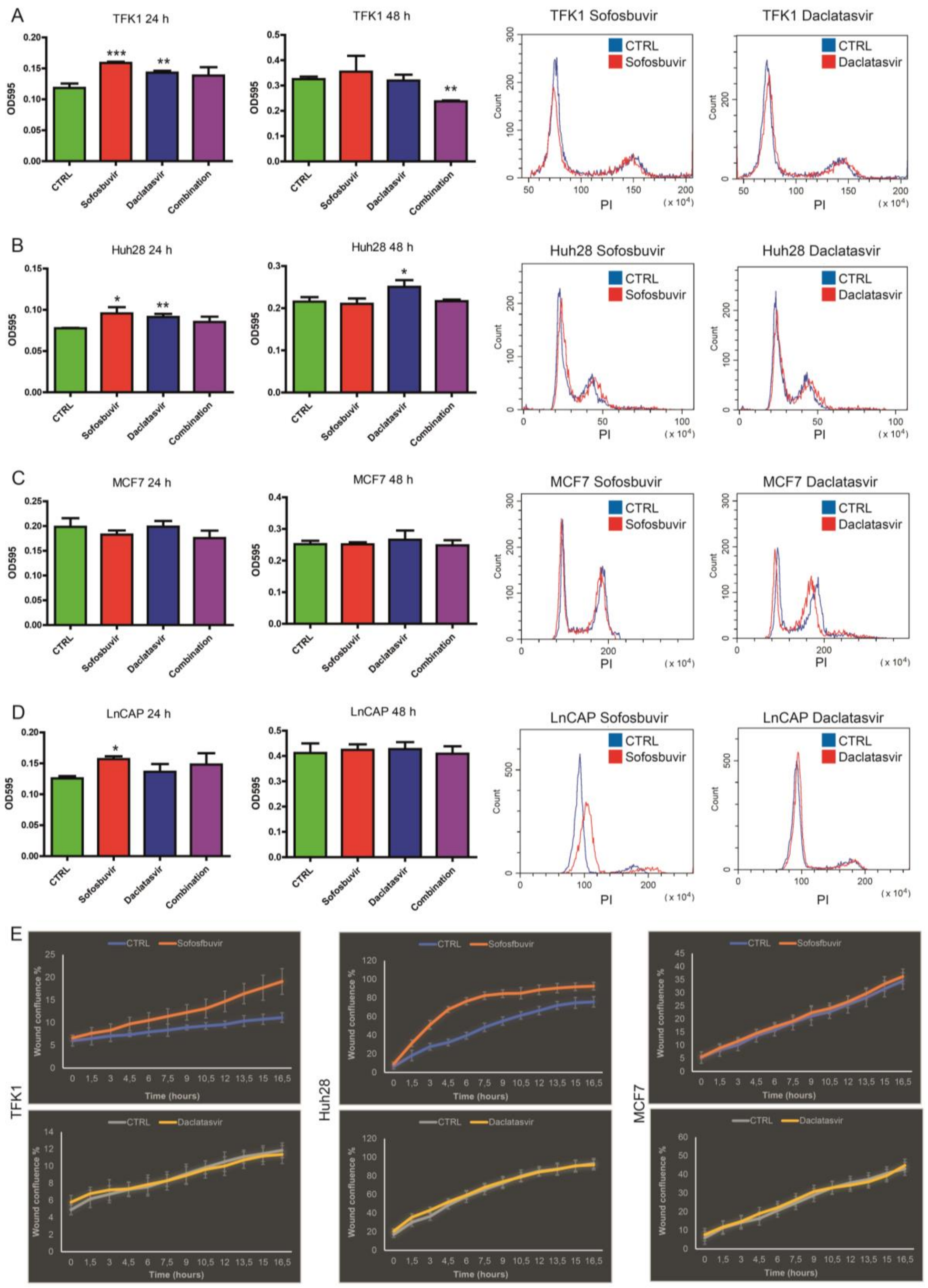

Figure S1. Effects of DAA on proliferation, cell cycle and migration of CAA-derived cell lines (TFK1 and Huh28 cells), breast cancer (MCF7) and prostate cancer (LnCAP) derived cell lines. Proliferation assay (24 and 48 h) and cell cycle analysis (24 h) of (**A**) TFK1, (**B**) Huh28, (**C**) MCF7, (**D**) LnCAP cell lines following treatment with sofosbuvir (4 μM), daclatasvir (10 nM) and combination of the two drugs. Control cells (CTRL) were treated with the same concentration of vehicle (DMSO). Student's *t*-test was used for comparison between each treatment and control cells. (**E**) Migration assay on the same non-HCC cell lines. * $p < 0.05$, ** $p < 0.01$, *** $p < 0.001$.

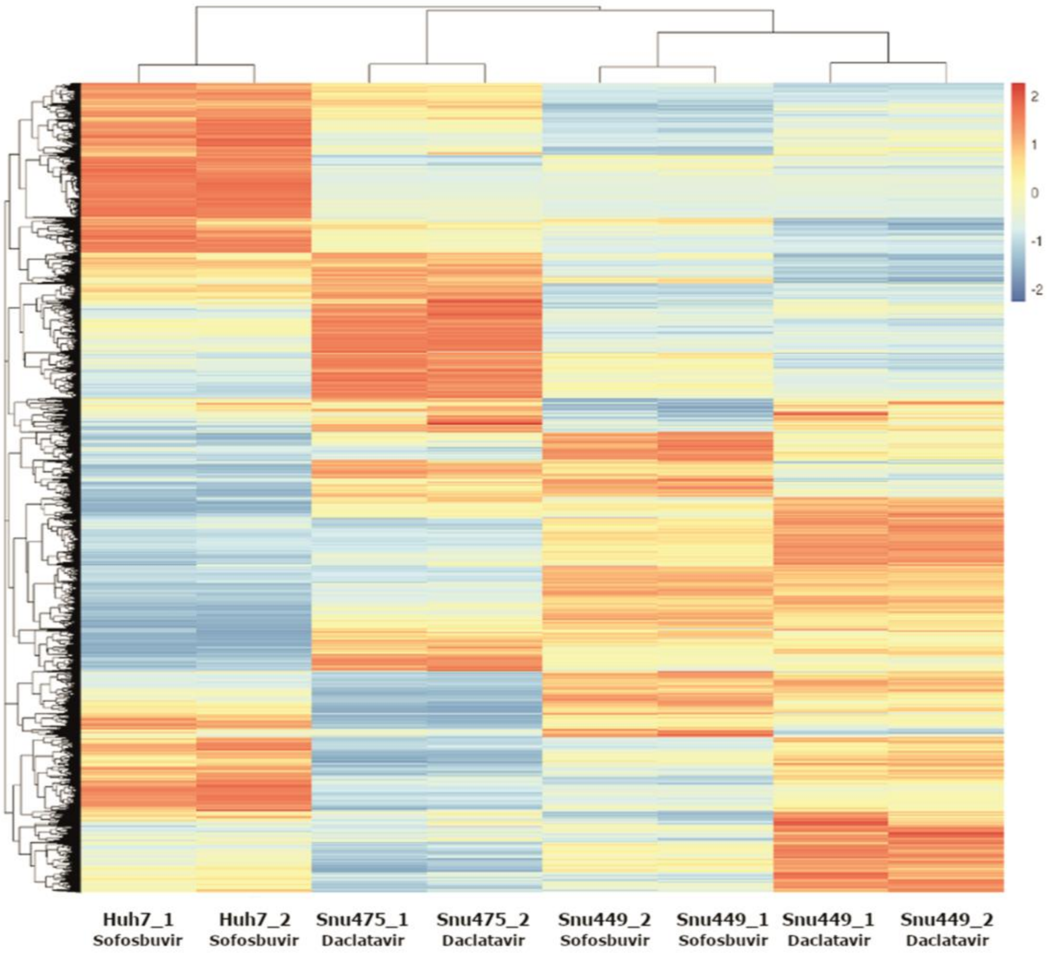

Figure S2. Heat map depicting transcriptional changes in DAA-treated HCC-derived cell lines. Four different clusters are highlighted in the column dendrogram corresponding to different cell lines and treatments.

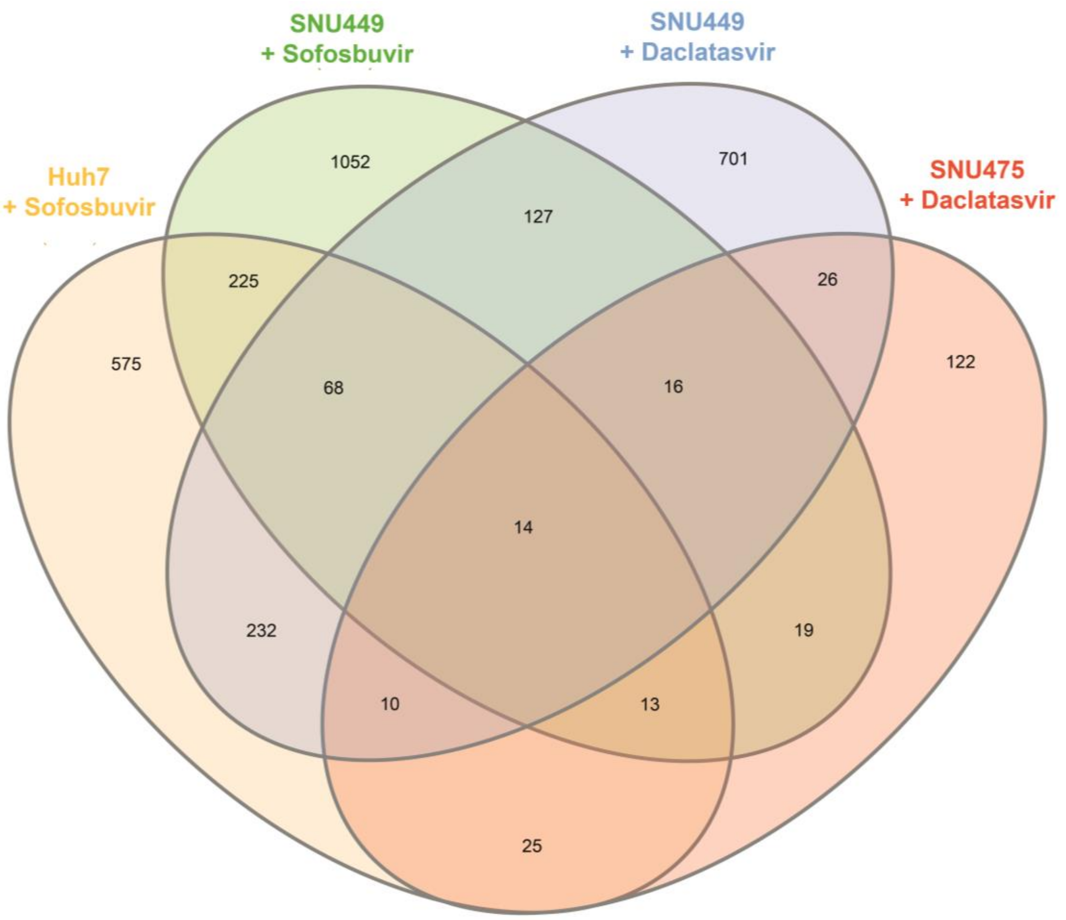

Figure S3. Venn diagram. The overlap of significantly modified genes in DAA-treated HCC-derived cell lines is displayed.

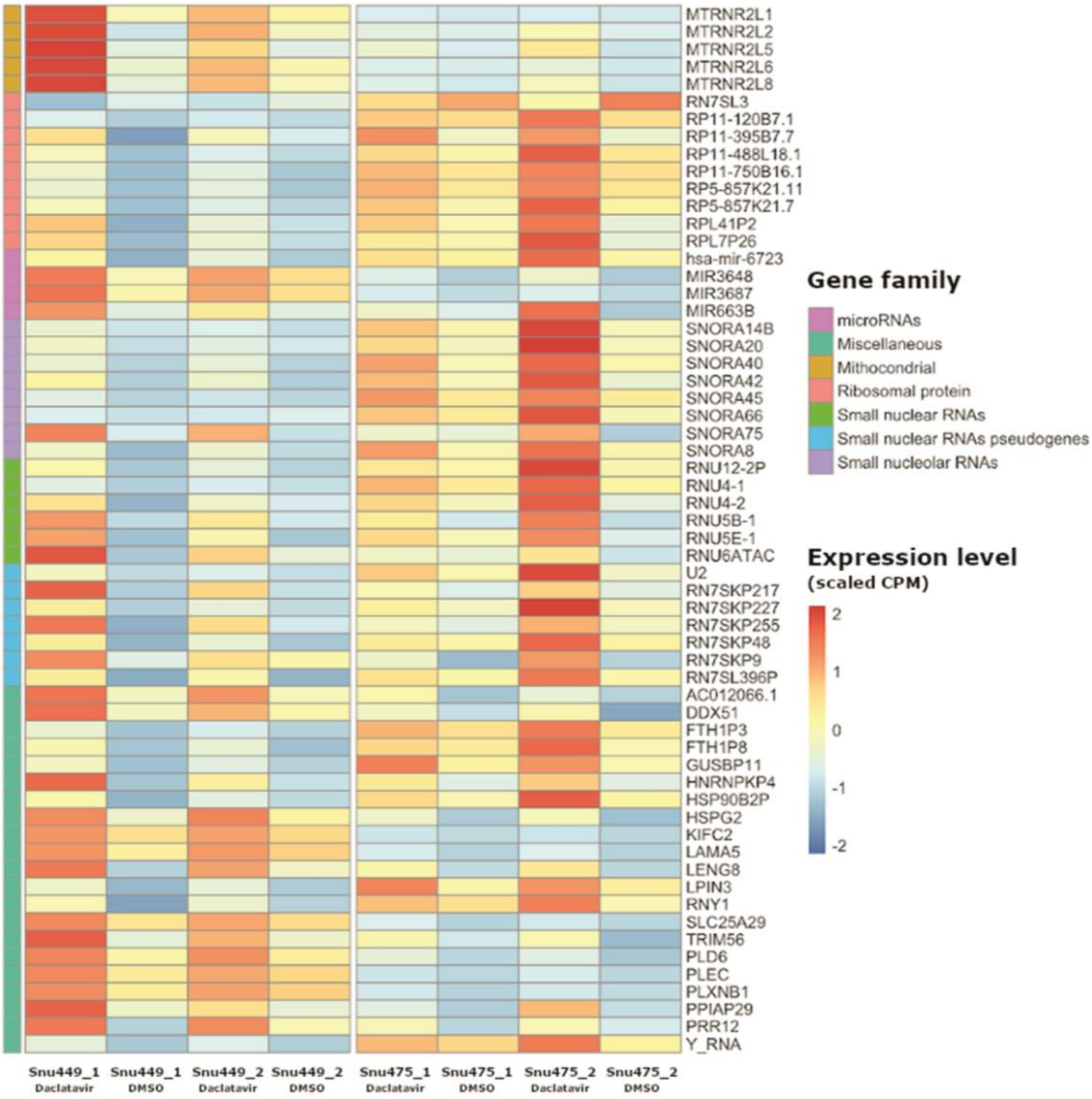

Figure S4. Overlapping genes modulated by daclatasvir in SNU449 and SNU475 cells. Gene families affecting cell proliferation are reported on the left.

References

1. Targett-Adams, P.; Graham, E.J.; Middleton, J.; Palmer, A.; Shaw, S.M.; Lavender, H.; Brain, P.; Tran, T.D.; Jones, L.H.; Wakenhut, F.; et al. Small molecules targeting hepatitis C virus-encoded NS5A cause subcellular redistribution of their target: insights into compound modes of action. *J. Virol.* **2011**, *85*, 6353–6368.
2. Gramantieri, L.; Pollutri, D.; Gagliardi, M.; Giovannini, C.; Quarta, S.; Ferracin, M.; Casadei-Gardini, A.; Callegari, E.; De Carolis, S.; Marinelli, S.; et al. MiR-30e-3p Influences Tumor Phenotype through MDM2/TP53 Axis and Predicts Sorafenib Resistance in Hepatocellular Carcinoma. *Cancer Res.* **2020**, *80*, 1720–1734.
3. Fornari, F.; Milazzo, M.; Galassi, M.; Callegari, E.; Veronese, A.; Miyaaki, H.; Sabbioni, S.; Mantovani, V.; Marasco, E.; Chieco, P.; et al. p53/mdm2 feedback loop sustains miR-221 expression and dictates the response to anticancer treatments in hepatocellular carcinoma. *Mol. Cancer Res.* **2014**, *12*, 203–216.
4. Giovannini, C.; Baglioni, M.; Toaldo, M.B.; Cescon, M.; Bolondi, L.; Gramantieri L. Vidatox 30 CH has tumor activating effect in hepatocellular carcinoma. *Sci. Rep.* **2017**, *7*, 44685.

© 2020 by the authors. Licensee MDPI, Basel, Switzerland. This article is an open access article distributed under the terms and conditions of the Creative Commons Attribution (CC BY) license (http://creativecommons.org/licenses/by/4.0/).
